# Evaluation of Single Nucleotide Polymorphisms (SNPs) Associated with Genetic Resistance to Bovine Paratuberculosis in *Marchigiana* Beef Cattle, an Italian Native Breed

**DOI:** 10.3390/ani13040587

**Published:** 2023-02-07

**Authors:** Piera Mazzone, Antonella Di Paolo, Linda Petrucci, Martina Torricelli, Sara Corneli, Carla Sebastiani, Marcella Ciullo, Martina Sebastianelli, Silva Costarelli, Eleonora Scoccia, Fiorella Sbarra, Federica Gabbianelli, Giovanni Chillemi, Alessio Valentini, Giovanni Pezzotti, Massimo Biagetti

**Affiliations:** 1 Istituto Zooprofilattico Sperimentale dell’Umbria e delle Marche “Togo Rosati”, Via Salvemini 1, 06126 Perugia, Italy; 2 Azienda Sanitaria Unica Regionale Marche, Area Vasta 2, Servizio di Igiene degli Allevamenti e delle Produzioni Zootecniche, 60127 Ancona, Italy; 3 A.N.A.B.I.C. Associazione Nazionale Allevatori Bovini Italiani Carne, Strada del Vio Viscoloso 21, San Martino in Colle, 06132 Perugia, Italy; 4 Department for Innovation in Biological Agro-Food and Forest Systems (DIBAF), University of Tuscia, 01100 Viterbo, Italy

**Keywords:** bovine paratuberculosis, *Mycobacterium avium* ssp. *paratuberculosis*, MAP, cattle, gamma-interferon test, IFN-γ, polymorphisms, SNP, local breeds, genetic resistance

## Abstract

**Simple Summary:**

*Mycobacterium avium* ssp. *paratuberculosis* (MAP) is the causative agent of paratuberculosis (PTB), chronic enteritis of ruminants spread worldwide. PTB is, by now, considered a conditioned disease, depending on both environmental and genomic factors. PTB susceptibility seems to be related to some genes coding for immune regulators involved in the cell-mediated response during infection: genetic markers, particularly single nucleotide polymorphisms (SNPs), have been investigated in several studies, and different candidate genes have been highlighted as associated with PTB resistance/susceptibility. The aim of this preliminary study was to verify, for the first time in a local beef cattle breed, known as *Marchigiana*, an association between MAP infection status and SNPs in candidate immune-genes. Specifically, in a MAP-infected herd, IFN-γ tests, ELISA, qPCR, and cultures were performed, for a follow-up range of 3–6 years, on 112 bovines to evaluate the state of MAP infection. Animals with positive results for at least one test were considered infected. DNA samples of bovines with a known MAP status were analyzed to assess the presence and the genotypic frequency of SNPs in genes encoding for gamma-interferon *(BoIFNG)*, interleukin receptor 10 *(IL10RA)*, interleukin receptor 12 *(IL12RB2)*, and toll-like receptors *(TLR1, TLR2, TLR4)*. For the *IL10RA* and *IL12RB2* genes, relevant differences in genotypic frequencies among the considered cattle groups were observed. For all the investigated candidate genes, SNP genotypes that have been associated with PTB resistance in the literature, were found more frequently, suggesting potential genetic resistance traits in the *Marchigiana* breed.

**Abstract:**

*Mycobacterium avium* ssp. *paratuberculosis* (MAP) is the causative agent of paratuberculosis (PTB), a widespread chronic enteritis of ruminants. The progression of the infection depends on the containment action of innate and cell-mediated immunity (CMI), and it is related to environmental and genetic factors. In particular, PTB susceptibility seems to be associated with specific genes coding for immune regulators involved in the cell-mediated response during the infection. The aim of this preliminary study was to verify, in Italian beef cattle, an association between MAP infectious status and the presence of single nucleotide polymorphisms (SNPs) in candidate genes. To the best of our knowledge, this is the first investigation conducted on a native beef cattle breed, known as *Marchigiana*, reared in Central Italy. The present research, based on a longitudinal study, aimed to identify and correlate phenotypic and genetic profiles characteristic of the subjects potentially able to contrast or contain PTB. In a MAP-infected herd, ELISA, IFN-γ tests, qPCR, and cultures were performed at a follow-up, occurring within a period ranging from three to six years, to evaluate the individual state of infection. Animals testing positive for at least one test were considered infected. DNA samples of 112 bovines, with known MAP statuses, were analyzed to verify an association with SNPs in the genes encoding gamma-interferon *(BoIFNG)*, interleukin receptor 10 (*IL10RA*), interleukin receptor 12 *(IL12RB2),* and toll-like receptors *(TLR1, TLR2, TLR4).* Regarding statistical analysis, the differences among target genes and pairs of alleles in the analyzed groups of animals, were evaluated at a significance level of *p* < 0.05. For *IL10RA* and for *IL12RB2* genes, relevant differences in genotypic frequencies among the considered cattle groups were observed. For all candidate genes studied in this investigation, SNP genotypes already associated with PTB resistance were found more frequently in our population, suggesting potential resistance traits in the *Marchigiana* breed.

## 1. Introduction

Paratuberculosis (PTB), or Johne’s disease (JD), is an infectious disease of ruminants caused by *Mycobacterium avium* subsp. *paratuberculosis* (MAP) and is characterized by a chronic granulomatous enteritis affecting farmed ruminants and wild species [1,2]. Clinical manifestations of the disease include persistent or intermittent diarrhea, decreased production, progressive weight loss, and often eventual death [3,4]. Infected animals can spread MAP through feces, and live bacteria can survive for a long time in pastures [5,6,7] representing a potential risk not only to other animals but also to humans [8,9]. In particular, for more than a century, it has been thought that MAP may be involved in the etiopathogenesis of human Crohn’s disease (CD), a chronic inflammatory bowel disease (IBD) characterized by transmural inflammation and granuloma formation [10,11,12]. Recently, other diseases have been associated with MAP, such as sarcoidosis, Blau syndrome, type 1 diabetes, Hashimoto’s thyroiditis, and multiple sclerosis [13,14,15,16,17,18]. In addition, MAP can contaminate food for human consumption, such as dairy and meat products, milk [19,20], and water [9,21]. 

Concerning animal husbandry, PTB infection can result in substantial economic losses, as it causes a decline in milk production [22,23], weight loss, infertility, and early culling of cows [24,25]. To date, PTB is widespread worldwide, and although several countries have undertaken control and certification programs, the prevalence of infected farms is rapidly increasing. PTB prevalence rates are estimated, globally, to be between 7% and 55% [26,27]; in Italy, the rate exceeds 50% [28]. Transmission occurs via the oro-fecal route during the first months of the calf’s life, following the ingestion of contaminated colostrum, milk, or feces [4,29]. The animal can then become persistently infected; however, the onset of the clinical form of PTB does not appear until 2–3 years of age, and it is strongly conditioned by farm management, the virulence of the strain and the individual animal’s immune system efficiency [30,31,32]. In the early stages of infection and the latency period, the containment action of cell-mediated immunity (CMI) plays an essential role [33]. The efficiency of this response determines the animal’s greater or lesser susceptibility to PTB in relation to environmental factors, as well. 

Different factors can influence the progression of infection, such as the host’s age, nutritional status, infection pressure, and the virulence of the pathogen. However, these factors alone cannot explain the extreme variations in disease outcomes [34]. Therefore, PTB can be defined as a conditioned disease, the infection is contracted at a very young age, but the transition from infected to affected animal is not obvious to all individuals, demonstrating a possible disease tolerance [35,36]. Indeed, only the animals “losing the battle against infection” become affected or diseased, thus showing clinical symptoms [34,35,37].

Several studies also suggest the existence of host genetic components in susceptibility or resistance to PTB, and investigations have focused on a set of genes encoding for immune-regulators involved in the cell-mediated response [34,38]. In particular, some association studies highlighted the correlation between PTB and polymorphisms in genes encoding for toll-like receptors (TLRs) [34,39,40,41,42]. TLRs belong to the pattern recognition receptor (PRR), a class of cellular receptors of the innate immune system [43], that are involved in the organism’s defense, and are capable of recognizing pathogen associated molecular patterns (PAMPs), and the typical structural profiles of bacteria, viruses, and fungi. TLRs’ recognition and interaction with PAMPs trigger the antigen-induced signal transduction pathway and activate transcription factors that regulate the expression of pro-inflammatory cytokines and chemokines, thus producing the inflammatory response. It has been observed that mutations in *TLR* genes, particularly single nucleotide polymorphisms (SNPs), are the primary cause of reduced pathogen recognition, contributing to increased susceptibility to some infections, and interfering with the immune response [44]. In human pathology, mutations in *TLR2* and *TLR4* genes can cause increased susceptibility to infections such as tuberculosis, malaria, acute rheumatic fever, urinary tract infections, and CD [39,45]. Indeed, mutations in *TLR1* and *TLR4* genes have been documented in the literature to cause a decreased response against bacterial cell wall components, such as lipopeptides and lipopolysaccharides, that are highly present in mycobacteria [39].

In cattle, 10 different types of TLRs [1,2,3,4,5,6,7,8,9,10], each with a specific function, have been identified. In particular TLRs 1, 2 [43,46], and 4 [39] are thought to be involved in the recognition of mycobacteria, including MAP [39,40,42]. After the ingestion of MAP, TLRs located on the surface of intestinal immune cells (i.e., antigen-presenting cells (APC)) bind the bacterial cell, thus inducing the expression of cytokines as interleukin IL-12, which are capable of promoting the differentiation of naive T cells into T helper 1 (Th1) cells. These cells, in turn, secrete gamma-interferon (IFN-γ), which is responsible for macrophage activation. After mycobacterium recognition, in the early stage of the infection, the secretion of pro-inflammatory cytokines, such as IFN-γ and IL-12, is essential for containing the MAP infection [47]. Additionally, in the later stage of the infection, T helper 2 (Th2) cells begin to secrete anti-inflammatory cytokines such as IL-10. These cytokines hamper macrophage activation, reducing the cytokines secreted by Th1 cells and promoting the humoral response [48]. This stage of infection can mark the transition from the latent to the clinical form of the disease. Therefore, the genes coding for these cytokines and their receptors have also been investigated in several studies [38,49]. In particular, SNPs associated with resistance/susceptibility to PTB have also been reported in the genes investigated in the present study, *IFN-γ*, *IL10RA,* and *IL12RB2* [40,41,42].

As widely known, during the latent stage of JD, infected animals spread MAP in feces before showing any clinical signs, representing an important source of infection for other animals in the herd [4,5,6]. Therefore, early diagnosis of the infection is important for the prompt removal of infected individuals that shed MAP in the environment to prevent the spread of JD. Several diagnostic tests, based on the direct and indirect detection of MAP, have been developed [29,50,51]. Currently, the *intra vitam* diagnosis of PTB is based on methods that assess the humoral immune response through serological testing, and direct detection of MAP from feces is carried out using cultures and PCR [52,53]. Unfortunately, these approaches only allow for detection among subjects with advanced stages of PTB infection, i.e., animals that already shed MAP or subjects in which the mycobacterium has already overcome the barriers of innate and CMI thus exhibiting seroconversion [35]. The initial host response against MAP infection is mediated by a Th1-type response, characterized by the production of IFN-γ and other pro-inflammatory cytokines [47]. This stage of infection can be highlighted by the IFN-γ test, an assay that reveals the CMI response established in subjects infected with or exposed to MAP [35,37,54]. In the IFN-γ test, the amount of the cytokine is detected in infected animals after T lymphocyte stimulation with purified protein derivatives (PPDs) extracted from mycobacterial cultures. In cattle, the IFN-γ test was developed for bovine tuberculosis (bTB) diagnosis [55,56,57]; however, in recent years, it has also been applied to PTB diagnosis [37,58], in particular using experimental PPD extracted from MAP cultures, namely Johnin (PPDJ), similar to that produced at the Istituto Zooprofilattico Sperimentale dell’Umbria e delle Marche “Togo Rosati” [59]. In the present study, to characterize the phenotype of the animals enrolled in the investigation, in addition to the traditional tests for PTB diagnosis, we also used IFN-γ tests, to detect infected but not diseased animals. Within the phenotyped groups, we performed genotypic characterizations, evaluating the distribution of genotype frequencies in candidate genes in a local cattle breed, named *Marchigiana*. 

In this regard, literature has further documented, also through whole-genome sequencing, different susceptibility levels among breeds of sheep [60], goats [61], deer [62], and cattle, where some breeds have shown to be resistant to MAP infection [34,63,64,65]. Italy represents one of the countries with higher cattle breed diversity, with more than 30 recognized local cattle breeds [66,67]. The typical biodiversity of native breeds is widely recognized by the European Community and the Food and Agriculture Organization (FAO), which are promoting the enhancement and preservation of the genetic variability present among the different European and Italian local cattle breeds [68]. While the European cattle gene pool is mainly derived from *Bos taurus taurus*, some Italian beef breeds such as *Chianina*, *Romagnola*, and *Marchigiana*, belonging to the so called “Podolian group” [69] and show a mixed origin of *B. t. taurus* and *B. t. indicus* ancestries [66,70,71]. In particular, the *Marchigiana* breed, the object of the present study, is a beef breed derived from the more ancient breeds *Chianina* and *Romagnola* [66,69,71]. It is assumed that native ancient breeds, being less selected over the years than breeds improved for productive traits, may have retained “diseases resistance genetic traits” [67]. In this regard, local cattle breeds have demonstrated both higher adaptation to the local environmental conditions and reduced susceptibility to many important infectious diseases [68].

On this basis, our preliminary study was conducted on the *Marchigiana* cattle breed, a typical local beef breed reared in Central Italy, in order to assess PTB resistance genetic traits, and verify the presence of SNPs in candidate genes encoding for target cytokines and their receptors, that are involved in the disease. The ultimate goal of the research, based on a longitudinal study, was the identification and correlation of phenotypic and genetic profiles characteristic of those individuals potentially able to contrast or contain MAP infection.

## 2. Materials and Methods 

### 2.1. Herds and Animals Identified for the Study

*Marchigiana* cattle from a bTB officially free (OF) herd, who had previous PTB positivity, were enrolled in the trial. At first, various farms were screened and monitored, however, the investigation was then focused only on a *Marchigiana* cow-calf beef farm, with breeding cows of high genetic value, that were older than 36 months. In this herd, farmer collaboration was guaranteed and the cows could be followed for a long time. Furthermore, in the herd, there were two bulls, used for natural mating, that were screened with a six-year follow-up. Additionally, the remaining animals in the farm, consisting of calves destined to be sold as breeding stock, were followed for at least 3 years.

No ethical approval was required because sampling was carried out concurrently with the periodic tests required by the Italian National Health Programs [72,73,74] and with the requests of the breeders for voluntary health controls for PTB, provided by the Italian National Guidelines [75]. 

### 2.2. Assessment of MAP Infection Status Using Traditional Methods 

In order to define the animals phenotypes and the MAP infection status, in a *Marchigiana* cattle herd, with previous serological positivity for PTB, 112 animals (aged from 2 to 10 years) were tested at least once per year and at a follow-up ranging from three to six years. Particularly, the animals were subjected to:

- a serological assay, ELISA test for PTB from blood serum (IDVet^®^-Grabels, France; IDEXX^®^-Westbrook, ME, USA) in accordance with the manufacturer’s instructions;
- molecular analysis, qPCR of IS900 target gene for the direct detection of MAP from feces, validated in fast mode [76,77,78]; and
- a MAP culture, on selective solid media, according to the OIE/WOAH Terrestrial Manual [58].

Regarding the interpretation of the results, animals found to have a positive result for at least one of the tests (serological and/or isolation and/or qPCR) were considered to be PTB-positive subjects.

### 2.3. Assessment of Cell-Mediated Immunity (CMI) Parameters

For the assessment of CMI parameters, heparinized blood samples from each animal, divided into aliquots of 1 mL, were respectively stimulated in vitro with:

- “Phosphate Buffered Saline” (PBS) without specific antigens;
- Bovine PPD and Italian Avian PPD (produced at IZSUM);
- Johnin PPD (produced at IZSUM); and
- Mitogen (BOVIGAM^®^ Pokeweed Mitogen-Thermofisher Scientific, Waltham, MA, USA) for the lymphocyte viability check.

After 18–22 h of incubation, plasma was collected and IFN-γ was detected with ELISA kit (BOVIGAM^®^-Thermofisher Scientific).

Gamma interferon assay outcomes were interpreted by considering the difference between the optical density (OD) values of PPDs(obtained after lymphocyte stimulation) and the basal OD value, using the cut-off value provided by the Bovigam^®^ kit (if PPDA or PPDJ—PBS > 0.1 = MAP infection), as described by Corneli et al., 2021 [37].

### 2.4. Phenotypic Categorization 

Based on the results obtained from the traditional methods, the animals were classified into three phenotypic groups:

- group 1: healthy, uninfected cattle, which were always negative for the ELISA, qPCR, and IFN-γ assays;
- group 2: healthy but MAP-infected cattle, with positivity for the IFN-γ test but always negative for the ELISA and qPCR assays; and
- group 3: PTB affected cattle, with positivity for at least the ELISA and/or qPCR from feces and/or the MAP culture, regardless of the IFN-γ test results.

The within-herd PTB prevalence was estimated at around 1.8%. Out of 112 analyzed subjects, 50 of them had negative results for the ELISA, IFN-γ test, and qPCR from feces (group 1). A total of 57 subjects were healthy but infected, with negative outcomes for the ELISA and qPCR from feces but positive outcomes for the IFN-γ assay (group 2). Finally, five animals (group 3) were PTB positive; in particular, five tested positive on the ELISA, and one of them also tested positive on the qPCR from feces and the MAP culture. 

### 2.5. SNPs Analysis for TLR-1, 2, 4, INF-γ, IL-10R, and IL-12R Genes 

Genomic DNA was extracted from whole blood using the High Pure PCR Template Preparation Kit (Roche Life Science, Mannheim, Germany), following the manufacturer’s instructions. 

Target genes and polymorphic sites were accurately selected from the literature (Table 1). For the *TLR4* gene sequence (446 bp), about 100 ng of extracted DNA was used as a template in PCR amplification and the optimal reaction concentration of the primer forward and reverse set of Mucha et al. [39] was 400 nM. PCR protocols were optimized with the following thermal cycling profile: an initial step of denaturation of 94 °C for 5 min and 35 cycles at 94 °C for 60 s, 62 °C for 45 s, and 72 °C for 60 s, and a further elongation step at 72 °C for 10 min. 

*TLR4* amplicons were controlled on 1.5% agarose gel electrophoresis containing Midori Green Advanced DNA Stain (Nippon Genetics Europe GmbH, Düren, Germany) and PCR products were purified with QIAquick^®^ PCR Purification Kit (Qiagen, Hilden, Germany), according to the manufacturer’s instructions. The quality and quantity of PCR products were assessed using a Biophotometer, (Eppendorf^®^, Hamburg, Germany), measuring the absorbance at 260, 280, and 230 nm and the relative ratio.

Sequencing reactions were performed, in both directions, using BrilliantDye™ Terminator Cycle Sequencing Kit v3.1 (NimaGen BV, Nijmegen, The Netherlands) according to the manufacturer’s instructions. Sequencing reactions were run in a 3500 Genetic Analyzer (Thermo Fisher Scientific). All sequences in the FASTA format were aligned to *Bos taurus TLR4* mRNA, (GeneBank accession number: NM_174198.6) Electropherograms were checked at each investigated mutation point to locate and discriminate heterozygous peaks. SNPs presence was assessed with BioEdit v7.2.5 software [79], using the ClustalW algorithm and was also confirmed using Unipro UGene software [80], to detect putative de novo polymorphisms.

The other target genes were analyzed in service by LGC Biosearch Technologies (Queens Road, Teddington, Middlesex, UK) with KASP™ (kompetitive allelic-specific PCR) SNP genotyping technology. 

### 2.6. Statistical Analysis

In order to evaluate whether there were statistically significant differences between the most represented phenotypic groups, a comparison among animals presenting the polymorphisms of interest for each investigated gene was carried out using Pearson’s chi-square, and considering the different allelic pairs of group 1 (healthy subjects) versus those of group 2 (healthy but infected subjects). Z-tests were performed for all target genes to compare the number of animals with genotypes related to each SNP by comparing the proportions of the two groups. The differences were considered significant at *p* < 0.05. The analysis was performed using Stata software v.11.2 (StataCorp LCC, Lakeway, TX, USA).

## 3. Results and Discussion

In our investigation, 50 animals, although exposed to MAP infection, were followed for a long period of time (from three years up to six years), and were always negative for the IFN-γ test, the ELISA, and the qPCR from feces (group 1). On the other hand, 57 animals only showed reactivity to the IFN-γ test against Avian PPD and Johnin, thus representing animals that have an acquired “immunological memory” against MAP (group 2). In addition, the remaining 5 animals of the herd, tested positive on the ELISA with one of them having positive results for the qPCR and MAP culture; still, they never showed clinical signs. For this reason, animals from group 3 were considered affected but not diseased, which is in accordance with the Whittington “JD case-definition” [54]. Thus, this category, not representing an “extreme phenotype”, was not included in the statistical analysis. These animals could be subjects that are susceptible to the infection but not necessarily to the disease. Therefore, we can assume that the phenotypes derived from the studied population reflect the characteristics of animals that resist or contain MAP infection, not manifesting PTB in a clinical form.

**Figure 1:**
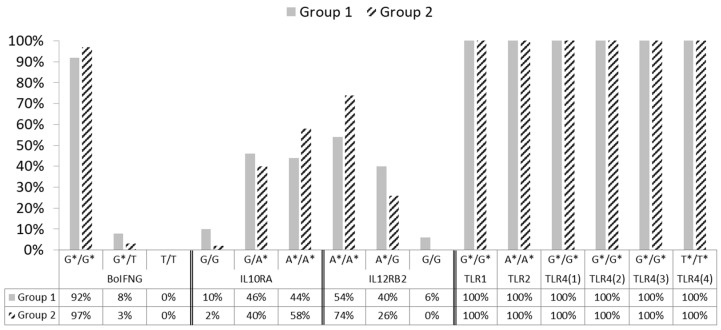
Histogram plot highlighting genotype frequency differences, between groups 1 and 2, for target gene polymorphisms investigated in the study; * Alleles associated with PTB resistance.

Regarding the candidate genes investigated in our preliminary study, Table 2 and Figure 1 report the distribution of genotype frequencies for each polymorphism, of the *BoIFNG*, *IL10RA*, *IL12RB2*, *TLR1*, *TLR 2* and *TLR4* genes found in the group of healthy subjects (group 1) and the group of infected ones (group 2). For *IL10RA*, there were four SNP variants (rs42395522, rs42395524, rs42395525, and rs42395526) considered by Verschoor [41]; however, since a strong linkage disequilibrium (LD) exists among polymorphisms, we decided to address the analysis for only one SNP (rs42395522) variant (Table 1). 

As reported in Table 2 and Figure 1, wild-type genotypes of the *BoIFNG*, *IL12RB2,* and all *TLRs* genes (which, in PTB susceptibility studies, are never associated with disease status) [34,39,40,42] were more frequent in our population. 

It is relevant to highlight that the wild-type G/G genotype of the *IL10RA* gene, which is associated with a high probability that the carrier animal is MAP infected and/or affected [41], occurred at a very low frequency in our population (Table 2; Figure 1). 

Conversely, the mutated A/A genotype of the same gene (44% for group 1 and 58% for group 2) and the heterozygous ones (46% for group 1 and 40% for group 2), which are associated with PTB resistance in literature [41], are mostly represented in our animals.

Table 2 and Figure 1 report the distribution of genotype frequencies for each of the SNPs of the target genes only in the two most represented phenotypic groups: 1 and 2. However, statistically significant differences between groups 1 and 2 were not observed in the candidate genes, except for the *IL12RB2* gene (A/A *p* = 0.03), which despite being present in each group, had a higher frequency in group 2 (54% for group 1 and 74% for group 2).

Regarding the phenotypic groups, it has to be considered that the specificity and sensitivity of diagnostic assays are influenced by the stage of MAP infection and by the individual host response. For instance, ELISA has lower sensitivity in the early stages than in the late stages of PTB. On the other hand, fecal cultures and qPCR from feces are strictly dependent on the intermittent and transient fecal MAP shedding of cattle [54]. These features make the identification of infected animals, and thus, the interpretation and agreement among the genetic association studies of JD difficult [34].

Furthermore, as already known, there is a substantial difference between PTB-affected animals and healthy animals but MAP infected animals [54], as highlighted by the evaluation of CMI parameters in particular, the IFN-γ test adopted in this study [37]. The IFN-γ positive animals are characterized by an immune system potentially capable of containing MAP infection and, for this reason, they could never become a MAP-shedder during their lifetime. Actually, our longitudinal study focused on this category because the ability of these animals to control the progression of infection could potentially represent the so-called PTB “resilient” or “resistant” subjects. The correlation between the immunity parameters analysis and the assessment of particular genetic traits and markers, such as SNPs in candidate genes with a key role in PTB pathogenesis, finally allows for the definition of susceptible/resistant animals.

It has to be considered that the comprehension of the mechanisms and genetic loci of resistance and disease tolerance to infectious agents in asymptomatic individuals is currently very limited [36]. However, it has been reported that the presence of polymorphisms in the *TLR1*, *2*, and *4* genes can affect the efficiency of the bovine immune system in containing MAP infection [34,39,45]. Likewise, SNPs detected in the genes of cytokines and their receptors seem to play a crucial role against PTB infection [40,41,42]. Additionally, an interesting study conducted by Koets et al. [46], provided evidence that, in cows with a susceptible *TLR2* haplotype, the clinical phase of PTB was more severe and started earlier as a consequence of inadequate innate and CMI responses. 

In this study, the distribution of genotype frequencies in the SNPs of candidate genes seems to confirm the same outcomes obtained in the phenotypic groups. In fact, genotypes at the polymorphic sites most represented within the negative and healthy but infected groups corresponded to the genotypes that, in the literature, are associated with subjects that are “not susceptible to PTB” and are, therefore, probably resistant. Conversely, the genotypes at polymorphic sites that are associated, in the literature, with susceptibility to PTB [34], in our population, were low or absent, as in the case of *BoIFNG* gene, (Table 2; Figure 1).

Finally, considering that the second group represents the IFN-γ test positive animals, which for the entire follow-up period never became positive for traditional PTB diagnostic tests, we can assume that these subjects could be those that, even if MAP infected, can successfully contain the disease. Thus, these subjects could represent the phenotype to be further investigated in future PTB resistance studies. 

## 4. Conclusions 

Up until now, several studies have been conducted to identify host-specific genetic factors that are predictive of MAP susceptibility; however, more research about the functional effects of SNPs and the downstream effects of protective Th1-type responses is needed. 

To the best of our knowledge, this is the first genetic investigation carried out on a local beef cattle breed, known as *Marchigiana*, which has been less selected over the years than breeds improved for productive traits. Like other native breeds, *Marchigiana* cattle are probable carriers of disease resistance genetic traits.

On this basis, and considering the genetic profiles found in this investigation, our preliminary results are promising. Thus, we are planning to utilize a more large-scale approach through a genome-wide association study (GWAS), on the *Marchigiana* breed and other Italian ancient beef cattle breeds, such as *Chianina*, in the perspective of future potential adoption of significant and robust genetic markers in MAS (marker-assisted selection) plans, allowing for the valorization of a native or ancient breed. 

## Figures and Tables

**Table 1:** Single Nucleotide Polymorphisms (SNPs) of candidate genes selected for this study.

Gene	Chr	SNP		Reference	Year
*TLR1*	6	658 G > A	missense	Mucha et al. [39]	2009
*TLR2*	17	2038 A > G	missense	Mucha et al. [39]	2009
*TLR4^1^*	8	892 G > Y	missense	Mucha et al. [39]	2009
*TLR4^2^*	8	895 G > A	missense	Mucha et al. [39]	2009
*TLR4^3^*	8	1165 G > A	missense	Mucha et al. [39]	2009
*TLR4^4^*	8	1167 T > C	missense	Mucha et al. [39]	2009
*BoIFNG*	5	2781 G > T	missense	Pinedo et al. [40]	2009
*IL10RA*	15	984 G > A *	silent	Vershoor et al. [41]	2010
*IL12RB2*	3	−511 ** A > G	promoter	Pant et al. [42]	2011

Chr: chromosome; TLR4^1^: 892 G > Y polymorphism; TLR4^2^: 895 G > A polymorphism; TLR4^3^: 1165 G > A polymorphism; TLR4^4^: 1167 T > C polymorphism; Y: C or T nitrogen base. *: ID variant (coordinate of polymorphism location at chromosome locus) = rs42395522; **: the minus sign means that the single nucleotide polymorphism is localized on the promoter region, upstream of the gene.

**Table 2:** Genotype frequencies of each single nucleotide polymorphism (SNP) for candidate target genes.

Group	Genotype Frequencies								
	BoIFNG	IL10RA	IL12RB2	TLR1	TLR2	TLR4^1^	TLR4^2^	TLR4^3^	TLR4^4^
**Group 1** **n = 50**	**G/G** 46/50 (92%)	G/G 5/50 (10%)	**A/A** * 25/47 (54%)	**G/G** * 48/48 (100%)	**A/A** 50/50 (100%)	**G/G** 50/50 (100%)	**G/G** 50/50 (100%)	**G/G** 50/50 (100%)	**T/T** 50/50 (100%)
	**G**/T 4/50 (8%)	G**/A** 23/50 (46%)	**A/**G * 19/47 (40%)	**G/**A * 0/48 (0%)	**A/**G 0/50 (0%)	**G/**Y 0/50 (0%)	**G/**A 0/50 (0%)	**G/**A 0/50 (0%)	**T/**C 0/50 (0%)
	T/T 0/50 (0%)	**A/A** 22/50 (44%)	G/G * 3/47 (6%)	A/A * 0/48 (0%)	G/G 0/50 (0%)	C/C, T/T 0/50 (0%)	A/A 0/50 (0%)	A/A 0/50 (0%)	C/C 0/50 (0%)
**Group 2** **n = 57**	**G/G** 55/57 (97%)	G/G * 1/55 (2%)	**A/A** 42/57 (74%)	**G/G** * 54/54 (100%)	**A/A** * 56/56 (100%)	**G/G** 57/57 (100%)	**G/G** 0/57 (100%)	**G/G** 0/57 (100%)	**T/T** 0/57 (100%)
	**G**/T 2/57 (3%)	G**/A** * 22/55 (40%)	**A/**G 15/57 (26%)	**G/**A * 0/54 (0%)	**A/**G * 0/56 (0%)	**G/**Y 0/57 (0%)	**G/**A 0/57 (0%)	**G/**A 0/57 (0%)	**T/**C 0/57 (0%)
	T/T 0/57 (0%)	**A/A** * 32/55 (58%)	G/G 0/57 (0%)	A/A * 0/54 (0%)	G/G * 0/56 (0%)	C/C, T/T 0/57 (0%)	A/A 0/57 (0%)	A/A 0/57 (0%)	C/C 0/57 (0%)
**Group 1** **+** **Group 2** **n = 107**	**G/G** 101/107 (94%)	G/G * 6/105 (6%)	**A/A** * 67/104 (64%)	**G/G** * 102/102 (100%)	**A/A** * 106/106 (100%)	**G/G** 107/107 (100%)	**G/G** 107/107 (100%)	**G/G** 107/107 (100%)	**T/T** 107/107 (100%)
	**G**/T 6/107 (6%)	G/**A** * 45/105 (43%)	**A/**G * 34/104 (33%)	**G/**A * 0/102 (0%)	**A/**G * 0/106 (0%)	**G/**Y 0/107 (0%)	**G/**A 0/107 (0%)	**G/**A 0/107 (0%)	**T/**C 0/107 (0%)
	T/T 0/107 (0%)	**A/A** * 54/105 (51%)	G/G * 3/104 (3%)	A/A * 0/102 (0%)	G/G * 0/106 (0%)	C/C, T/T 0/107 (0%)	A/A 0/107 (0%)	A/A 0/107 (0%)	C/C 0/107 (0%)

Alleles associated with PTB resistance are reported in bold; group 1: ELISA, qPCR and IFN-γ negative subjects; group 2: ELISA and qPCR negative and IFN-γ positive subjects; Y: C or T nitrogen base; * subjects with a not defined genotype were excluded from the analysis; TLR4^1^: 892 G > Y polymorphism; TLR4^2^: 895 G > A polymorphism; TLR4^3^: 1165 G > A polymorphism; TLR4^4^: 1167 T > C polymorphism; n: number of animals.

## Data Availability

Not applicable.
